# Mint3 as a Potential Target for Cooling Down HIF-1α-Mediated Inflammation and Cancer Aggressiveness

**DOI:** 10.3390/biomedicines11020549

**Published:** 2023-02-14

**Authors:** Noritaka Tanaka, Takeharu Sakamoto

**Affiliations:** Department of Cancer Biology, Institute of Biomedical Science, Kansai Medical University; 2-5-1 Shin-machi, Hirakata 573-1010, Osaka, Japan

**Keywords:** Mint3, hypoxia, HIF-1, MT1-MMP

## Abstract

Hypoxia-inducible factor-1α (HIF-1α) is a transcription factor that plays a crucial role in cells adapting to a low-oxygen environment by facilitating a switch from oxygen-dependent ATP production to glycolysis. Mediated by membrane type-1 matrix metalloproteinase (MT1-MMP) expression, Munc-18-1 interacting protein 3 (Mint3) binds to the factor inhibiting HIF-1 (FIH-1) and inhibits its suppressive effect, leading to HIF-1α activation. Defects in Mint3 generally lead to improved acute inflammation, which is regulated by HIF-1α and subsequent glycolysis, as well as the suppression of the proliferation and metastasis of cancer cells directly through its expression in cancer cells and indirectly through its expression in macrophages or fibroblasts associated with cancer. Mint3 in inflammatory monocytes enhances the chemotaxis into metastatic sites and the production of vascular endothelial growth factors, which leads to the expression of E-selectin at the metastatic sites and the extravasation of cancer cells. Fibroblasts express L1 cell adhesion molecules in a Mint3-dependent manner and enhance integrin-mediated cancer progression. In pancreatic cancer cells, Mint3 directly promotes cancer progression. Naphthofluorescein, a Mint3 inhibitor, can disrupt the interaction between FIH-1 and Mint3 and potently suppress Mint3-mediated inflammation, cancer progression, and metastasis without causing marked adverse effects. In this review, we will introduce the potential of Mint3 as a therapeutic target for inflammatory diseases and cancers.

## 1. Introduction

Mammalian cells generally require molecular oxygen to produce ATP as an energy source through oxidative phosphorylation (OXPHOS) in mitochondria. However, certain types of cells, such as cancer cells and macrophages, are constitutively dependent on ATP production through an alternate metabolic pathway called glycolysis, and this phenomenon is known as the Warburg effect [1,2]. Hypoxia-inducible factors (HIFs), especially HIF-1α, are transcription factors that have roles in immune response, angiogenesis, cell growth, metabolism, and cell death [3]. Owing to the multiple roles of HIF-1α, its activity is associated with various diseases (e.g., inflammatory and metabolic diseases and cancer proliferation and metastasis) [4,5,6,7]. Therefore, the proper regulation of HIF-1α is essential for maintaining healthy metabolism and homeostasis. As HIF-1α is expressed ubiquitously in various tissues, the direct inhibition of HIF-1α can cause simultaneous adverse effects. One member of the Munc-18-1 interacting protein (Mint) family, Mint3, has been identified as an indirect activator of HIF-1α. Its effect is limited because its role as an HIF-1α activator requires the expression of membrane type-1 matrix metalloproteinase (MT1-MMP), which is limited to specific cells, such as macrophages, cancer cells, and fibroblasts [8,9,10,11]. Herein, we summarize the key roles and influences of Mint3.

## 2. Overview of HIF Proteins

As a key factor associated with the adaptation of cells to low-oxygen environments, HIFs are known to play crucial roles as transcription factors [12,13]. HIF proteins include three alpha subunits (HIF-1α, HIF-2α, and HIF-3α) and one beta subunit (HIF-1β); they are heterodimers composed of one of the three alpha subunits and HIF-1β. HIF alpha subunits are stably expressed under hypoxic conditions, whereas HIF-1β is stably expressed independent of oxygen [14]. All HIF proteins share a basic domain and a dimerization domain within the N-terminal. However, alpha subunits but not beta subunits possess an N-terminal transactivation domain (NAD) in the C-terminal, and only HIF-1α and HIF-2α have the C-terminal transactivation domain (CAD) [15,16]. These structural differences among the four types of HIF subunits lead to differences in protein stability and transcriptional activity regulation. The HIF subunits also show variations in their expression locus and target genes. While HIF-1α is ubiquitously expressed in human tissues and upregulates the expression of glycolysis-related genes, HIF-2α is expressed in specific tissues, such as tumor vascular cells and macrophages, and promotes erythropoietin and iron metabolism [17,18,19]. HIF-3α has an important role in adipogenesis, and its gene expression is regulated by HIF-2α activity [20]. The stability of all alpha subunits is conditionally regulated by oxygen concentration; however, HIF-1β is stably and ubiquitously expressed. HIF requires the heterodimerization of alpha and beta subunits for its activation. Therefore, alpha subunits (especially HIF-1α) are responsible for regulating HIF activity by switching between aerobic and hypoxic conditions through the upregulation of glycolysis-related genes. The protein stability and activity of HIF-1α are suppressed through two mechanisms during normoxia: (1) the proteasomal degradation of HIF-1α through NAD modification and (2) the enzymatic inhibition of HIF-1α through CAD modification. Under normal oxygen conditions, HIF-1α is hydroxylated at the proline residues P402 and P564 by proline hydroxylase domain (PHD) proteins (mainly PHD2), and these modifications are recognized by von Hippel Lindau (VHL), a component of the E3 ubiquitin ligase complex, resulting in proteasome-dependent degradation [21,22,23]. Because PHDs use oxygen for the hydroxylation of HIF-1α, the destabilization of HIF-1α by PHDs occurs only when oxygen is sufficient [24]. The transcriptional activity of HIF-1α is achieved through its stabilization during hypoxia and its interaction with the transcriptional co-activator cAMP response element binding protein (CREB)-binding protein (CBP)/p300 protein. The factor inhibiting HIF-1 (FIH-1) protein functions as an asparaginyl hydroxylase and contains two core domains: a catalytic domain, which is homologous to the cupin protein family, and a dimerization domain in the C-terminal region [25,26]. Dimerized FIH-1 is involved in the hydroxylation of HIF-1α at the N803 residue within CAD and interferes with the interaction between HIF-1α and the CBP/p300 proteins [25,27,28,29]. Additionally, FIH-1 recruits VHL to HIF-1α by binding with its N-terminal region, which induces the destabilization of HIF-1α [28]. With these two different hydroxylase-dependent mechanisms, cells regulate the protein stability and activity of HIF-1α and adapt to a low-oxygen environment by switching the mechanism of ATP production from OXPHOS to glycolysis. 

## 3. Mechanisms of Mint3-Mediated HIF-1 Activation

### 3.1. Mint3 Indirectly Upregulates HIF-1α Activity through Its Interaction with FIH-1

Mint3, also known as amyloid-beta A4 precursor protein-binding family A member 3, is a novel protein that positively regulates HIF-1α [30]. The Mint family consists of three isoforms: Mint1, Mint2, and Mint3 (also known as APBA1, APBA2, and APBA3; X11, X11-like, and X11-like2; and X11α, β, and γ, respectively). Mint1 and Mint2 bind with Munc-18-1, which is involved in neural homeostasis [31]. Mint3 shows ubiquitous expression in various tissues, with the lowest level in the testis, according to Okamoto and Sudhof [32]. Its intracellular localization is mainly at the Golgi apparatus where it interacts with furin [33]. Mint1-3 were originally identified as neural proteins that interact with the YENPTY motif within amyloid precursor proteins (APPs), which are conserved in the cytoplasmic region of APP and modulate their activities [32,34,35]. All three Mint isoforms share highly conserved structures in their C-terminal region, which comprise one phosphotyrosine-binding (PTB) domain followed by tandem PDZ domains (PDZa and PDZb). Through these two domains, Mint3 functions as an adaptor protein. With its PTB domain, Mint3 binds to proteins such as furin, N-terminal EF-hand calcium binding protein 3 (NECAB3), and Rab6 [32,33,36,37]. Moreover, PDZ binds with proteins such as breakpoint cluster region protein (Bcr) [38]. Amyloid precursor protein and Arf GTPase interact with both the PTB domain and the PDZb (but not PDZa) domain [38,39].

**Figure 1:**
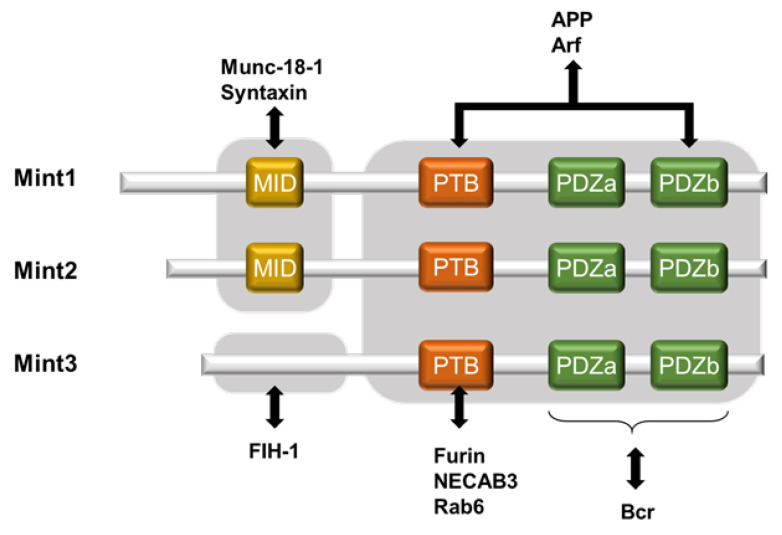
The schematic structures of Mint proteins with their binding partners. Mint family comprises well-conserved C-terminal region with one phosphotyrosine-binding domain (PTB) and tandem PDZ domains (PDZa and PDZ b); differential N-terminal region with Munc-18-1 interacting site (MID) only for Mint 1 and 2; FIH-1 binding site for Mint3. FIH-1—Factor inhibiting HIF-1, NECAB3—N-terminal EF-hand calcium binding protein 3, APP—amyloid precursor protein, and Arf—ADP-ribosylation factor (Arf)-GTPase.

However, only Mint1 and Mint2 retain the N-terminal region, which contains Munc-18-1 interacting domain; because Mint-3 lacks the domain, it is unable to interact with Munc-18-1 [32] (Figure 1). Additionally, a recent structural analysis of Mint3 revealed that its N-terminal region is intrinsically disordered [40]. Thus, the sequences of the N-terminal region of Mint3 differ substantially from those of the other Mint proteins, and, as a result, Mint3 has divergent binding partners at its N-terminal region. Notably, the sequences in each Mint family member are highly conserved among species [32]. Our investigation, involving a yeast two-hybrid screening with FIH-1 as a bait protein, revealed that the N-terminal region could interact with FIH-1 and that neither Mint1 nor Mint2 showed such an affinity, indicating that FIH-1 selectively binds with Mint3. Further investigation revealed that the N-terminal region of Mint3, especially amino acid residues G78–G88, and the dimerization domain of FIH-1 are required for interactions between these two proteins [40]. As a result, Mint3, especially its N-terminal region, competes with HIF-1α as an FIH-1, leading to an indirect interruption of HIF-1α hydroxylation at N803 by FIH-1. A comprehensive investigation to clarify the key mechanism of FIH-1 binding with Mint3 was performed, and the results revealed that at least two amino acid regions of the dimerized FIH-1 (78–88 and 101–110) are required [41]. In addition, the phosphorylation of the T5 and S7 amino acid residues within Mint3, mediated by the mammalian target of rapamycin complex 1 (mTORC1) but not by mTORC2, is required for HIF-1α activation [42]. These studies described the “trapping” of FIH-1 by Mint3 in the trans-Golgi network through the interaction of Mint3 with FIH-1 and furin [33] with its N-terminal and C-terminal regions, respectively, which allows HIF-1α to escape catalytic inhibition by FIH-1. 

### 3.2. Factors That Support Mint3-Mediated HIF-1α Activation

Although Mint3 is ubiquitously expressed, its effect on HIF-1α regulation is limited to specific regions where MT1-MMP is expressed simultaneously, such as fibroblasts, macrophages, and cancer cells. MT1-MMP, also known as MMP14, is a member of the MMP family. The MMP family comprises 23 members, most of which are composed of well-conserved structures. The extracellular domains include the pro-domain, catalytic (CAT) domain, which binds with zinc ions, and hemopexin (HPX) domain; in addition, a transmembrane region and an adjacent cytoplasmic region are present [10]. Among the 23 MMPs, 6 are recognized as membrane-anchored MMPs, which are further classified into two types as follows: 1) MT1-MMP (MMP14), MT2-MMP (MMP15), MT3-MMP (MMP16), and MT5 (MMP-24) are transmembrane types, which conserve the cytoplasmic region, and 2) MT4-MMP (MMP-17) and MT6-MMP (MMP-25) are glycosylphosphatidylinositol-anchored types, which lack the cytoplasmic region [10,43]. MT1-MMP is initially expressed in an inactive state (pro-MMP); however, it switches to the active form upon proteolytic processing in the pro-domain by furin [44]. MT1-MMP is the first transmembrane MMP type to be associated with the upregulation of invasion and the metastasis of cancer cells [45]. This is somewhat convincing because the most important substrates of MT1-MMP are the extracellular matrix (ECM) molecules, especially type-1 collagen, with MT1-MMP catalyzing various extracellular proteins, including secreted proteins (e.g., growth factors, cytokines/chemokines, and collagens) and cell surface proteins (e.g., adhesion molecules and transmembrane receptors) [46,47]. When MT1-MMP is expressed in macrophages or cancer cells, these cells exhibit invasive features such as the invasion of macrophages into the basement membrane and cancer metastasis [9,48].

**Figure 2:**
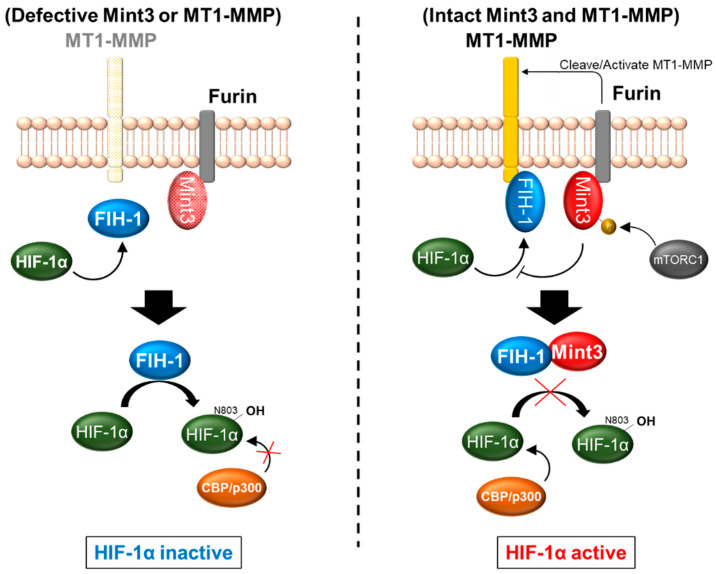
Mint3 indirectly activates HIF-1α. Mint3 binds with factors inhibiting HIF-1 (FIH-1) upon furin-mediated activation of membrane type 1-matrix metalloproteinase (MT1-MMP) followed by FIH-1 defected in the ability to suppress HIF-1α. Mint3 progress HIF-1α-mediated phenomenon including glycolysis, acute inflammation, and cancer progression and metastasis.

Apart from the functions of MT1-MMP as a proteinase, it also plays an important role in HIF-1α activation by supporting the Mint3–FIH-1 interaction. Twenty amino acids within the cytoplasmic tail (CPT) of MT1-MMP enable FIH-1 to have direct contact with MT1-MMP, resulting in the inhibition of FIH-1′s suppression of HIF-1α activity [9,49]. In addition, this binding between MT1-MMP and FIH-1 is accomplished independently of the N-terminal region of MT1-MMP, which contains pro-domain, CAT, and HPX. Thus, this interaction leads to the binding of Mint3 and FIH-1, resulting in HIF-1α activation. Of note, the activation of HIF-1α mediated by the MT1-MMP–FIH-1–Mint3 axis only requires the CPT of MT1-MMP; hence, CPT that lacks affinity with FIH-1 is unable to activate HIF-1α [49]. This indicates that MT1-MMP activates HIF-1α independent of its N-terminal region. As Mint3 is mainly localized to the Golgi apparatus by binding with furin, MT1-MMP possibly catalyzes the interaction of Mint3 and FIH-1 by adhering FIH-1 to the Golgi apparatus, thus, keeping it in close contact with Mint3 instead of diffusing to the cytoplasm [33,49]. Overall, Mint3 requires the coordinated expression of MT1-MMP to activate HIF-1α; therefore, the critical influence of Mint3 activity is restricted to loci with sufficient levels of MT1-MMP expression, such as in macrophages, cancer cells, and fibroblasts. In addition to MT1-MMP, NECAB3 is reportedly involved in Mint3-mediated HIF-1α activation [37], indicating that other unidentified factors could affect the Mint3–FIH-1–HIF-1α axis. The cascade of HIF-1α activation through the axis of MT1-MMP, Mint3, and FIH-1 is summarized in Figure 2.

## 4. Mint3 Mediates Inflammatory Responses

Macrophages are the key players in innate immunity and rely on constant HIF-1α-mediated glycolysis regardless of oxygen conditions [4,50], which is dependent on Mint3 activity. Upon stimulation or infection by various pathogens, macrophages are involved in producing cytokines and chemokines, growth factors, and reactive oxygen species (ROS) during inflammation to protect the host. However, the overactivation of the immune system can rather be toxic due to septic shock [51]. Stimulation by lipopolysaccharides (LPS), which are a toxic component of the outer membrane of Gram-negative bacteria, causes septic shock. This toxicity is a consequence of a cytokine storm mediated by the glycolysis-dependent secretion of cytokines associated with increased motility and invasiveness of macrophages, which involve the Mint3–FIH-1–HIF-1α axis [52]. Macrophages gain “lethal force” by Mint3 expression under LPS-driven immune reaction.

In an immune response to influenza virus (IFV), Mint3 depletion efficiently improves influenza pneumonia by alleviating the production of cytokines and chemokines in macrophages (but not in dendritic cells) through two mechanisms: the upregulation of Adenosine 5′-monophosphate (AMP)-activated protein kinase (AMPK) α activity and the stabilization of IκB [53]. AMPK is activated through its phosphorylation on Adenosine 5’-triphosphate (ATP) starvation [54] and downregulates nuclear factor kappa B (NF-κB) activity [55]. The depletion of Mint3 downregulates glycolysis-mediated ATP production and activates AMPK and the subsequent inhibition of NF-κB. Concurrently, Mint3 deficiency leads to the glycolysis-independent stabilization of IκB, which causes the simultaneous inhibition of NF-κB. Although the precise mechanisms through which Mint3 deficiency causes IκB stabilization remain elusive, the hydroxylation of IκB by FIH-1 might regulate IκB stability [56]. However, Mint3 does not contribute to gaining immunity against IFV, as Mint3 depletion has no influence on the production of interferon (IFN)-α or anti-IFV antibodies [53].

Pyroptosis is a mechanism of programmed cell death that is associated with innate immunity and occurs when cells encounter intracellular bacterial infection. It was initially pointed out by Friedlander and was named by D’Souza to distinguish it from apoptosis [57,58]. Canonical pyroptosis signals involve the formation of inflammasomes, which mostly comprise Nod-like receptors (NLRs)/absent in melanoma 2 (AIM2); apoptosis-associated speck-like protein (ASC) as an adaptor protein; and the inflammatory caspase-1, whereas caspase-11/4/5 could serve as an inflammatory caspase in non-canonical pyroptosis. Inflammasomes process the pyroptosis-executing protein gasdermin D (GSDMD) and interleukin (IL)-1β/IL-18. *Listeria monocytogenes* (LM) is a common foodborne pathogen that causes zoonotic diseases in Western countries by infecting various types of cells. Upon innate infection by LM, the host operates a protective mechanism by clearing out the bacteria through the production of ROS and nitric oxide (NO) induced by the activation of pyroptosis [59,60,61,62]. However, deficient Mint3 protects mice against LM-derived lethality without affecting NO production and rather decreasing ROS production. As ROS inactivates caspase-1 by oxidization, the suppression of ROS production by Mint3 depletion stabilizes caspase-1 and activates pyroptosis [63]. Additionally, the formation of inflammasomes that comprise AIM2, NLRP3, and ASC are inhibited by glycolysis, Mint3 depletion activates pyroptosis by diminishing glycolysis and promoting the caspase-1-dependent processing of GSDMD and IL-18/IL-1β. Therefore, Mint3 depletion protects the host of LM by two mechanisms: (1) stabilization of caspase-1 by suppressing ROS production and (2) promoting the formation of inflammasomes by diminishing glycolysis. Although the mechanisms of the inhibition of ROS production in Mint3-depleted macrophages have not been elucidated, it could be mediated by the inhibition of HIF-1α and NF-κB, which plays a role in producing pro-oxidant genes, including Nicotinamideadenine-dinucleotide phosphate (NADPH) oxidase (*NOX2*) [53,64,65].

Inconsistent with these potential effects of Mint3 on the immune system, a previous report from the group of Huai [66] showed that Mint3 potentiates an antiviral response through the expression of IFN-β upon the induction of immune signals after viral infection or LPS stimulation through Toll-like receptors (TLRs) and retinoic acid-inducible gene-I–like receptors (RLRs) [67,68,69,70]. In this case, Mint3, with its PDZ domains within the C-terminal regions, serves as an adaptor protein for tumor necrosis factor receptor-associated factor 3 and enhances K63-linked polyubiquitination, which activates downstream TLR- or RLR-mediated signaling [71].

This indicates that Mint3 plays an important role in maintaining the basic functions of macrophages; however, once macrophages are stimulated by various pathogens, such as LPS, viruses, and bacteria, Mint3 generally acts as an enhancer of acute inflammation. Moreover, in the case of LM infection, Mint3 attenuates the death of “sick” macrophages, implying that the environment surrounding the macrophages remains infected. When the macrophages are kept “healthy”, Mint3 acts as a “housekeeper” as it is involved in ATP production by activating glycolysis; however, Mint3 can be toxic for the organisms once the immune signals are induced by the pathogens.

## 5. Role of Mint3 in Cancer Progression

The effects of Mint3 on the innate immune system may be applicable in cancers because Mint3 has the potential to increase cancer malignancy and metastatic features through its expression in inflammatory monocytes (IMs), which are defined with the following markers: Gr-1/Ly6C^+^, CD11b/CD115^+^ [72]. In addition, a relationship between Mint3 activity in cancer cells and cancer-associated fibroblasts (CAFs) has been previously reported [11,73,74]. Because Mint3 has key roles in enhancing cancer progression and metastasis by upregulating HIF-1α, which is also related to the tumor microenvironments, it could be an attractive target for cancer treatment, as tumor-microenvironments-related factors are gaining more attraction, which is described as one of the important hallmarks of cancers [75]. In this section, we summarize the influence of Mint3 on cancer progression through its expression in Ims, cancer cells, and CAFs.

### 5.1. Impact of Mint3 Activity in Cancer Cells

Solid tumors in human bodies are often exposed to low-oxygen environments due to the disadvantage in physical distance from the blood vessels and obtaining sufficient oxygen. In order to adapt to such circumstances, cancer cells tend to retain high HIF-1α activities and gain malignancies. Although the depletion of Mint3 in the host of xenograft models has no effects on tumor progression, its depletion in cancer cells results in HIF-1α suppression, which is associated with downregulated glycolysis, angiogenesis, and anti-proliferative effects. These Mint3-related features are commonly seen in the xenografts of various types of cancer cells (e.g., breast cancer, MDA-MB-231; fibrosarcoma, HT-1080; epidermoid carcinoma, A431; non-small cell lung cancer, A549; and urothelial carcinoma, RT-112) [11,73,74,76]. However, in pancreatic cancer cells, the depletion of Mint3 induces tissue-specific anti-proliferative effects in vitro and in vivo. This was mediated by the inhibition of *Skp2* transcription, which promoted G1/S transition in cell cycles and suppressed the induction of metastasis and stemness [73]. In addition, the Mint3-dependent expression of *Skp2* also involves the Mint3–FIH-1–HIF1α axis and is dependent on HIF-1α activity. Unlike in hypoxic conditions, pancreatic cancer cells show high expression and activity of HIF-1α-dependent *Skp2* in normoxic conditions. Consistent with the known features of Skp2 and HIF-1α as metastasis enhancers in various types of cancers [77,78,79,80], Mint3 promotes epithelial–mesenchymal transition in a HIF-1α- and Skp2-dependent manner with an enhancing effect on the expression of the Slug protein. A recent Mint3-related study on urothelial carcinoma also indicated that the depletion of Mint3 downregulates the invasion, migration, and proliferation of cancer cells, accompanied by factors associated with suppressed HIF-1α activity, such as transcription of HIF-1α-targeted genes and glycolysis [74]. 

### 5.2. Metastatic Ability of Cancer Cells Achieved through Mint3 Expression in IM

Using the PyMT breast cancer mouse model, which develops palpable breast cancers that metastasize to the lung, Qian et al. reported that IMs, which are characterized by Gr-1/Ly6C^+^, CD11b/CD115^+^ markers, play a key role in the lung metastasis of breast cancer in a C–C motif chemokine ligand 2 (CCL2)-dependent manner [81]. Other studies using xenograft mouse models of various types of cancers have shown that Mint3 plays an important role in this mechanism of metastasis [72,82]. Cancer cells and the surrounding stromal cells are the sources of CCL2, and they increase the number of IMs in the peripheral blood. Both individual depletion of Mint3 or HIF-1α in IMs leads to the decreased production of vascular endothelial growth factor (VEGF) to comparable levels. These effects of Mint3 or HIF-1α depletion on VEGF expression are consistent with the fact that VEGF is one of the target genes of HIF-1α [72,83]. Upon Mint3-dependent VEGF secretion by IMs, E-selectin expression in endothelial cells and vascular permeabilization of cancer cells are elevated [72]. Conclusively, IMs are recruited to the lung metastatic site in a HIF-1α- or glycolysis-dependent manner, which is driven by Mint3, to release vascular endothelial growth factor (VEGF), and this secretion of VEGF induces the expression of E-selectin in the epithelial cells in the lung, which enables the extravasation of cancer cells [72]. Only macrophages/monocytes work as metastatic inducers, and T cells or B cells do not possess similar functions. Notably, IMs secrete VEGF in accordance with the existence of cancer cells; however, its mechanism remains elusive.

### 5.3. Supportive Effect of Mint3 Expression in CAFs on Cancer Progression

CAFs are one of the factors that have a crucial role in promoting cancer malignancy through ECM remodeling, immune crosstalk, and metabolic effects [84]. These features of CAFs are achieved by functioning as a source of various secretory proteins, such as growth factors, cytokines, and exosomes, whereas the adherent molecules in CAFs could also increase tumor malignancy in a Mint3-dependent mechanism. Mint3 is also involved in the proliferation of cancer cells by regulating the expression of L1 cell adhesion molecule (L1CAM) in fibroblasts. L1CAM is a cell adhesion molecule that acts as a binding partner of heterodimeric integrins, such as α5β1, αvβ3, αIIbβ3, and αvβ5 [85,86], and triggers downstream signaling pathways, such as the MAPK and PI3K/Akt pathways [85,87]. Fibroblasts express L1CAM in a Mint3-dependent manner, which enables CAFs to promote direct contact with cancer cells expressing integrin α5β1. Therefore, Mint3 also plays a role in enhancing the proliferation of cancer cells through CAFs [11]. Because CAF-mediated ERK phosphorylation specifically occurs in the peripheral regions of breast cancers, which are surrounded by stroma, L1CAM is possibly involved in the local progression of breast cancers. The expression of Mint3 in CAFs is slightly upregulated by cytokines such as tumor necrosis factor-α and IL-1β. Indeed, CAFs express higher levels of MT1-MMP than normal fibroblasts do, which is also reflected in the activity level of Mint3 in fibroblasts. Further investigation is required to determine whether the activity level of Mint3 has an unlimited correlation with the expression level of MT1-MMP.

## 6. Therapeutic Efficacy of Targeting Mint3-Related Environments

In some cases, the activity of HIF-1α is toxic in terms of its ability to promote inflammatory diseases or cancer malignancies. Thus, HIF-1α has long been regarded as a beneficial therapeutic target. Many efforts have been made to develop HIF-1α inhibitors, but none have been successful, although some clinical trials are still underway. This is probably due to the ubiquitous expression of HIF-1α, which influences various factors. The first HIF-2α inhibitor, belzutifan (Welireg), was clinically approved for patients with VHL diseases and some types of cancers in 2021. Naphthofluorescein (Naph) was first identified as a potent Mint3 inhibitor. It effectively disrupts the protein interaction between Mint3 and FIH-1, leading to the suppression of HIF-1α activity with reduced expression of HIF-1α-targeting genes, glycolysis, and ATP production [76]. The inhibitory effect of Naph on Mint3 in inflammatory diseases effectively diminishes cytokine synthesis; upon LPS stimulation, this is well reflected in the increased survival of mice with LPS-induced inflammatory diseases. Moreover, Naph retains its inhibitory potential against chemotaxis of IMs toward CCL2–expressing cancer cells and the surrounding stromal cells, thereby reducing the expression of E-selectin in metastatic lung cancers without marked adverse effects. Notably, the pharmacological effect of Naph on metastasis is not affected in Mint3-deficient mice, which implies that Naph can suppress metastasis specifically by inhibiting Mint3 [76]. Conclusively, the pharmacological inhibition of Mint3 by Naph is effective against both immune-signal-related diseases and cancer progression with minimal adverse effects. The depletion of Mint3 potentiates the efficacy of a combined therapy of gemcitabine and paclitaxel, which is currently a standard regimen for pancreatic cancers. A combined therapy of gemcitabine and Naph reportedly has a synergistic effect against the progression of urothelial carcinoma cells in vivo. These preclinical outcomes indicate that Mint3 targeting could be a therapeutic intervention for pancreatic and urothelial cancers [73,74]. However, we cannot ignore the fact that Naph has low solubility in water and can also bind to furin, which could potentially result in unexpected outcomes [88].

## 7. Conclusions

**Figure 3:**
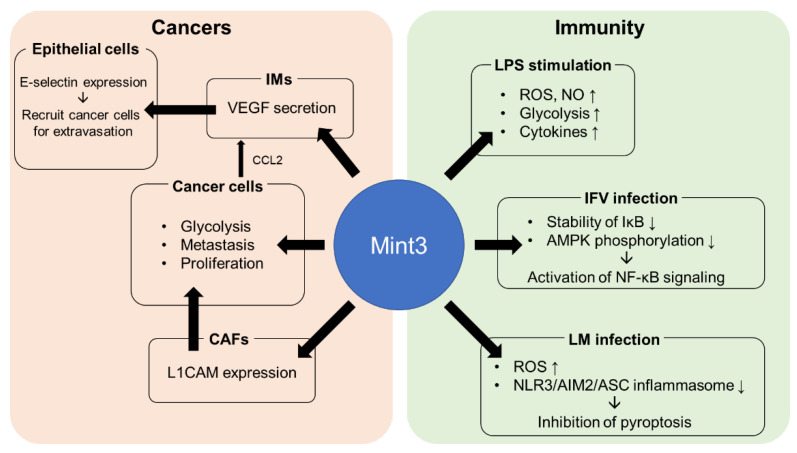
Summary of diverse effects of Mint3 on innate immune system and cancer progression. Mint3 mediates the production of cytokines/chemokines in various mechanisms depending on pathogens. In cancers, Mint3 promotes proliferation and metastasis with the involvement of inflammatory cells (IMs), cancer-associated fibroblasts (CAFs), or direct expression in cancer cells.

Mint3 inhibition effectively disrupts HIF-1α-mediated inflammations or cancer progressions. Although Huai et al. reported that Mint3 acts as a protective factor against inflammatory stimuli by enhancing IFN-β production associated with the positive feedback loop of Mint3 expression, Mint3 generally mediates biologically lethal effects by promoting acute inflammatory responses (Figure 3). Mint3 can promote the proliferation and metastasis of cancer cells and complicate cancer treatment by employing macrophages as invasion enhancers and CAFs as cell growth drivers [11,72]. In some cases, Mint3 directly promotes the proliferation and metastasis of cancer cells, such as pancreatic and urothelial cancer cells [73,74]. Therefore, Mint3 could be a potential therapeutic target for innate immune-response-related diseases and cancer. Because of the ubiquitous expression of HIF-1α and its influence on various factors related to biological maintenance, HIF-1α deficiency can cause various adverse effects. The direct targeting of HIF-1α is challenging as a result; thus, the indirect inhibition of HIF-1α by targeting Mint3 would potentially be beneficial. This is because Mint3 requires the cooperative expression of MT1-MMP, the expression of which is limited to specific areas, such as macrophages/monocytes, cancer cells, and endothelial tips, and the intact function of Mint3 is also limited to these areas. Mint3 knockout mice models of either acute inflammation upon stimuli by various pathogens or xenografts with various cancer cells showed significant improvement in the diseases. Naph, as a Mint3 inhibitor, inhibits the secretion of inflammatory cytokines and the proliferation and metastasis of tumors in vivo without causing marked adverse effects [74,76]. Therefore, Naph, a potential inhibitor of Mint3 as a therapeutic target, ought to be further explored. 

## Data Availability

Not applicable.
